# Conditions Modifying Results of Operations*Read before the North Texas Medical Association November, 1914.

**Published:** 1915-03

**Authors:** J. M. Inge

**Affiliations:** Denton, Texas


### Conditions Modifying Results of Operations.*

BY J. M. INGE, DENTON, TEXAS.

There exists no subject of more importance to be considered, especially by a large percentage of surgeons who are speedily building up or merging into a decided surgical practice, and many of whom are skillful technical surgeons, than conditions affecting results of operations, especially terminal results.

How are we to impress or warn the class of operators who are ever ready or inclined to urge immediate operative procedures without even stopping to properly consider the importance of a more accurate diagnosis or prognosis, even among those who in a way pretend to, but in a slipshod way make use of laboratory methods, than to remind them of such conditions in our papers and discussions before medical associations? Let it be understood we do not assume the rôle of an especial adviser or instructor, but do we not receive more lasting impressions and learn more valuable lessons from mistakes of our own making?

While the importance of the consideration of infection in relation to mortality cannot be overestimated, it has often resulted in overshadowing other important conditions, which, if left alone, bring a fatal issue, either directly or as a contributory cause.

Laboratory and research workers have given us ample evidence, based on experimental as well as clinical evidence, that exhaustion, which is the lack of functioning of fundamental organs, is a decided factor in surgical cases, enlarging the per cent of fatalities. The lack of functioning of such organs being due to disease processes, emotion, traumatism, etc., such unrecognized conditions often increased or decidedly developed to the extent of overwhelming the patient by the traumatism of the operative procedures. Acidosis, a condition ever fraught with danger, overcome under normal conditions by the natural functioning of the liver and adrenals, but when such organs are impaired so that they do not share in the neutralizing process of restoring the body fluids to their natural alkaline conditions, results in or portends serious conditions for the surgeon to meet.

As Crile has pointed out, the only remedy for such conditions is prevention by increasing the store of energy, and by stopping the expenditure of energy, and the consequent fabrication of acid. Such conditions are best treated by absolute quiet and rest by the

*Read before the North Texas Medical Association November, 1914.

administration of easily digestible nourishing diet, and the administration of an alkaline treatment. The consideration of the attitude of the patient to the ordeal of the operation has not usually provoked the serious thought its importance demands. The dread and worry in anticipation of a major operation with a large per cent of patients adds much to an unfavorable condition.

The surgeon whose clientele is so large, upon whose time demands are so exacting that he cannot consider the personality of his patient apart from the technique, impressing him with the idea that he is for the time his child to be manipulated with that thoughtful care that his helpless condition at the hands of his trusted benefactor demands, had best limit his work in order to add to his efficiency.

Of no less importance is the question of post-operative pain. In the sanitarium I administer about $\frac{1}{6}$ grain sulph. morphic and 1-150 grain hydrobromide hyoscin. In country homes, where room is limited and I must prepare for the operation almost in the presence of the patient, I usually give $\frac{1}{4}$ grain sulph. morphic and 1-100 grain hydrobromide hyoscin, and find after receiving this dose in thirty or forty minutes the patient will dose and take no notice of his environments, such as the usual display of instruments and dressings—will often not remember having been placed on the table—although he answers questions, and talks if questioned.

I have not depended on the scopolomine and morphine anesthesia in major surgery, to the exclusion of inhalation anesthesia, but one medium dose to precede the inhalation adds much to the prompt and efficient effect of the ether or chloroform as well as to the post-operative condition of the patient. The administration of local anesthesia or the practice of nerve blocking about the field of operation has been, with me, a great factor, not only in limiting the amount of the inhalation anesthetic, but serves to relax the abdominal muscles to that extent that the surgeon can handle and manipulate the viscera in a manner and with ease not to be compared without its use. As Crile has pointed out, he can go to the viscera instead of bringing it out of the cavity.

The prevention of post-operative gas pains and vomiting, the quiet rest for hours, the prevention of shock, the improvement of the condition of the circulatory system have combined to convince the author that its use marks an era for decided improvement of conditions in the practice of surgery.

As to diagnosis and prognosis in surgical cases, blood counts, microscopic findings and chemical analysis are not only not to be underestimated, but the surgeon who fails to make use of such methods must be relegated to a past age. Still, the doctor, with good surgical judgment, who has been a student of symptoms and conditions in the clinic and at the bedside, elicited by physical signs, including heart sounds, palpation inspection, his estimate of the blood pressure, will be found reasonably accurate, and must depend on such findings not only in many emergency cases, but in all classes of cases will find much to be gained by examination with the hands on the patient, rather than depend almost exclusively upon laboratory methods, as few modern surgeons seem inclined to do.

